# Prelamination of Neourethra with Uterine Mucosa in Radial Forearm Osteocutaneous Free Flap Phalloplasty in the Female-to-Male Transgender Patient

**DOI:** 10.1155/2016/8742531

**Published:** 2016-03-16

**Authors:** Christopher J. Salgado, Lydia A. Fein, Jimmy Chim, Carlos A. Medina, Stephanie Demaso, Christopher Gomez

**Affiliations:** ^1^Division of Plastic, Aesthetic, and Reconstructive Surgery, University of Miami Miller School of Medicine, Miami, FL 33136, USA; ^2^Department of Obstetrics and Gynecology, University of Miami Miller School of Medicine, Miami, FL 33136, USA; ^3^Department of Urology, University of Miami Miller School of Medicine, Miami, FL 33136, USA

## Abstract

Radial forearm free flap phalloplasty is the most commonly performed flap for neophallus construction in the female-to-male (FtM) transgender patient. Urological complications, however, can arise quite frequently and can prevent the patient from urinating in the standing position, an important postsurgical goal for many. Using mucosa to construct the fixed urethra and to prelaminate the penile urethra has been successful in reducing urologic complications, particularly strictures and fistulas. Until now, only buccal, vaginal, colonic, and bladder sites have been described as sources for these mucosal grafts. We present the successful use of uterine mucosa for prelamination of the neourethra in an FtM patient who underwent hysterectomy and vaginectomy at the prelamination stage of a radial forearm phalloplasty. Three months postoperatively, the patient was able to void while standing and showed no evidence of stricture or fistula on retrograde cystogram. These results suggest that uterine mucosa may be used for prelamination of the penile neourethra in patients undergoing phalloplasty.

## 1. Introduction

For many female-to-male (FtM) transgender persons, construction of a neophallus is a crucial culminating step in the gender transition. The goal of this surgery is to create a sensate, aesthetically pleasing phallus that can be used both for sexual intercourse and for micturition in the standing position. Though many techniques have been described, the radial forearm free flap remains the most common for phalloplasty due to its long, reliable vascular pedicle, multiple nerve innervations, and pliability [1].

Despite regarding it as the preeminent technique, urological complications remain the ubiquitous challenge of radial forearm phalloplasty. In the largest published series, 41% of patients suffered urological complications, mostly urethral strictures and fistulae; however, complications have also been reported as high as 80% [2, 3]. These sequelae particularly arise in the FtM patient because the natal female urethra must first be lengthened to build the pars fixa of the neourethra, which is subsequently anastomosed to the pars pendulans within the constructed radial forearm free flap. Buccal mucosa is commonly used for reconstructive urethroplasty because it is hairless, accustomed to a wet environment, and easy to harvest and it has a thick epithelium [4]. Thus, vaginal mucosa, which possesses properties similar to buccal mucosa, is often used to construct the pars fixa in the FtM patient [5, 6].

Prelaminating the penile neourethra in advance of flap transfer may reduce urologic complications when compared to the single-stage tube-within-a-tube technique for radial forearm flap creation [3, 6]. Split thickness skin grafts have been most commonly used for prelamination, but strictures and fistulas still arise, frequently at the junction of the pars fixa and pars pendulans. Given the historical success of reducing fistula and stricture rates in the fixed urethra with mucosa grafts, prelamination of the penile urethra with mucosa may be beneficial. In a series by Zhang et al., prelaminating the penile urethra with vaginal mucosa ahead of pedicled phalloplasty resulted in a stricture rate of only 4.5% and a fistula rate of 31.8%, of which nearly half healed spontaneously [5].

Unique to the FtM patient is the availability of his own uterine mucosa undergoing hysterectomy concomitantly with the first portion of a staged phalloplasty procedure. An option that has not been previously described is the use of uterine mucosa for prelamination of the penile neourethra. The aim of this case report is to describe the successful use of uterine mucosa for prelamination of the penile urethra within a radial forearm osteocutaneous free flap for phalloplasty in the FtM transgender patient.

## 2. Methods

A 20-year-old transgender male presented to University of Miami Center for Aesthetic and Corrective Genital Surgery for evaluation towards gender confirmation surgery (GCS). He was an appropriate candidate for phalloplasty based upon the World Professional Association for Transgender Health's Standards of Care [7]. He provided two letters from mental health professionals confirming his readiness for GCS. He was counseled regarding total abdominal hysterectomy (TAH), bilateral salpingo-oophorectomy (BSO), vaginectomy, and staged radial forearm osteocutaneous free flap phalloplasty with prelamination.

**Figure 1:**
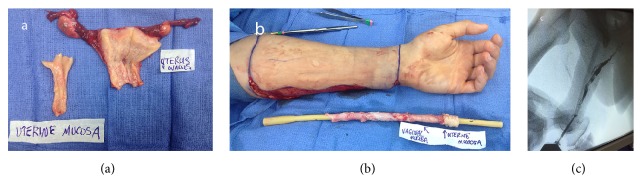
(a) Uterus and ovaries following hysterectomy and oophorectomy with portion of uterine mucosa resected for prelamination; (b) uterine and vaginal mucosa lining Foley catheter for prelamination of radial forearm flap; (c) retrograde cystourethrogram demonstrating patent neourethra.

After risks and benefits were explained to the patient, informed consent was obtained. The first stage of the procedure entailed exploratory laparotomy, TAH, BSO, vaginectomy, and urethral lengthening using an anteriorly based vaginal mucosa flap and labia minora tissues, followed by left radial forearm flap prelamination. The posterior vaginal mucosa was harvested as donor tissue for prelamination as was a piece of uterine mucosa from the remnant endometrial cavity of the excised uterus (Figure 1(a)).

Prelamination of the patient's eventual penile urethra was performed by grafting uterine and vaginal mucosa in a suprafascial plane of the left volar and ulnar forearm. This was done by first cleansing all mucosal grafts with a betadine and normal saline solution and then sewing the grafts around a 24-French Foley catheter construct exteriorizing the submucosal surface using a running, locking suture. The uterine mucosa was placed distally so that monitoring may be readily performed (Figure 1(b)). Placing this construct lengthwise in the subcutaneous forearm allowed for creation of a tubular graft, which would become the penile neourethra of the eventual phalloplasty. The patient was immobilized in a splint and in the immediate postoperative period showed no signs of infection. Irrigation of the prelaminated flap was performed twice daily beginning one week after surgery. To allow for irrigation of the entire prelaminated neourethra, holes were cut into the Foley catheter prior to sewing the mucosal grafts.

Approximately six weeks after the first stage flap prelamination, creation of the neophallus was performed by transferring the prelaminated construct as a radial forearm free osteocutaneous flap for inset over the denuded clitoris and lengthened urethra. Although allowing more time between stages may be favorable, we have found that six weeks is long enough to achieve successful wound healing and favorable results and is a time frame that is tolerable for our patients.

A urethral anastomosis was performed between the previously lengthened native urethra and the neourethra created within the flap with vaginal and uterine mucosa. We completed our phalloplasty with neurovascular microsurgical anastomoses, local tissue rearrangement to the genitalia, gracilis scrotoplasty, and coronoplasty. The patient was then transferred to surgical intensive care unit for standard free flap postoperative monitoring. After three weeks of hospitalization, during which transfer of split thickness skin graft to the forearm donor site over previously placed bovine collagen took place, the patient was discharged with both a suprapubic catheter and penile urethral catheter in place.

Two months following the second stage of phalloplasty, the patient was taken to the operating room for pericatheter retrograde cystourethrogram (RCUG) and cystoscopy.

## 3. Results

The RCUG demonstrated total urethral patency without evidence of strictures or fistulas (Figure 1(c)). Similarly, cystoscopy was performed without difficulty and the mucosa of the constructed urethra was confluent with the native urethra. A biopsy was taken of the distal-most aspect of the urethra and demonstrated endometrial tissue, as was expected given the original placement of the uterine mucosal graft. The penile Foley catheter was not replaced because urethral patency was established, and the suprapubic tube was clamped.

In a voiding trial, the patient was able to successfully void in the standing position through this neophallus. The patient was provided with a meatal dilator and given instructions for daily urethral meatus dilation to prevent distal stenosis of the neourethra.

## 4. Discussion

The ability to void while standing is a priority for most transgender men seeking phalloplasty, yet preventing urological complications associated with neourethra construction remains a challenge. Promising outcomes have been achieved by using mucosa for both urethral lengthening and prelamination of the neourethral conduit [6]. Within our patient population, using mucosa and prelamination has significantly decreased our urologic complications, including anastomotic fistulae.

While prelamination has predominantly been performed with skin grafting, mucosal grafts allow for greater homology between native urethra and the constructed components. Histologically, mucosa has nonkeratinized epithelium and therefore has greater similarity to the urethra than skin, which has a keratinized epithelium [5, 8]. There is also a greater chance of successful wound healing with mucosa, leading to less scar contracture at the recipient site [4].

Uterine mucosa is readily available if the patient is undergoing hysterectomy prior to phalloplasty in the same operative setting. Mucosa can be obtained whether the patient is undergoing abdominal, laparoscopic, or vaginal hysterectomy. This mucosa, particularly in its atrophic state, as it likely is in the FtM patient, has a nonkeratinized, glandular epithelium on a dense, fibrous stroma [9]. Both its lack of keratinization and its tensile strength make it an excellent candidate for construction of the penile urethra.

Other mucosal grafts may be limited or require additional surgical sites. Both buccal and bladder mucosal grafts are viable options, but introducing additional surgical sites can increase the risk of infection and other complications. Vaginal mucosa, while economic in that the excised mucosa would otherwise be discarded, may be insufficient if the patient has a small vaginal canal attributable to nulliparity and testosterone therapy. When adequate mucosa cannot be harvested, composite grafting of skin and mucosa is another option, but this may lead to worse outcomes than a graft comprised entirely of mucosa [6].

We have demonstrated that uterine mucosa may also be used to construct a patent, functional urethra with radial forearm phalloplasty. Though in this attempt we opted to prelaminate with both vaginal and uterine mucosa, the initial success suggests that uterine mucosa is a viable option to prelaminate the penile urethra in conjunction with vaginal mucosa, but quite possibly as the sole mucosal graft as well. However, further investigation is necessary.

The risk of developing endometrial cancer in the graft is low as this tissue is no longer exposed to estrogen following oophorectomy, and the patient's testosterone use may actually be protective [10]. This concern, however, led us to place the uterine mucosa at the distal-most aspect of the constructed phallus to allow for monitoring. If malignancy were to develop, resection would not significantly compromise the constructed phallus.

## 5. Conclusion

The addition of uterine mucosa as a viable option for prelamination of the penile neourethra provides a new strategy for potentially reducing urethral complications after phalloplasty by providing an additional source of mucosa for prelamination and allowing surgeons to avoid unnecessary operative sites as well as the use of composite grafts. Although more thorough investigation is needed, including longer follow-up and additional cases, the initial success of prelamination of the penile urethra with uterine mucosa in radial forearm free flap phalloplasty demonstrates a new technique in the arsenal of the surgeon performing FtM gender confirming surgery.
